# Slugs hide in the dark: Artificial light at night alters fitness and activity of dominant herbivores with consequences for ecosystem functioning

**DOI:** 10.1016/j.isci.2025.112770

**Published:** 2025-05-27

**Authors:** Vincent Grognuz, Flavienne Landolt, Julia Curty, Eva Knop

**Affiliations:** 1 Agroscope, Reckenholzstrasse 191, 8046 Zurich, Switzerland; 2 Department of Evolutionary Biology and Environmental Studies, University of Zurich, Winterthurerstrasse 190, 8057 Zurich, Switzerland

**Keywords:** Environmental science, Ecology, Zoology

## Abstract

Artificial light at night (ALAN) has emerged as a major source of disturbance for organisms by altering their behavior, physiology, and fitness, with consequences for ecosystem functioning. Yet, we still know relatively little about how it affects herbivores and the ecosystem function they provide. By experimentally illuminating not only captive individuals from two slug species but also entire wild communities, we tested whether nocturnal lighting alters circadian activity patterns, fitness parameters, feeding, and moving behaviors and whether this affects herbivory damage. In one of the two illuminated slug species, we found that ALAN reduced its activity at night, juvenile growth, and survival rate. At the community level, feeding activity of slugs was reduced in artificially illuminated sites, and this was related to reduced herbivory. We conclude that ALAN affects the fitness of slugs and disrupts their activity, with consequences for herbivory. This will most likely have indirect consequences for other ecosystem processes.

## Highlights

• Impact of artificial light at night (ALAN) is largely unexplored in terrestrial gastropods
• ALAN altered juvenile growth development and reduced survival rate of captive slugs
• Fewer wild slugs fed at sites with artificial night lighting than at unlit control sites
• Plants at lit sites showed less herbivory damage than those at unlit control sites

## Graphical abstract

**Figure:**
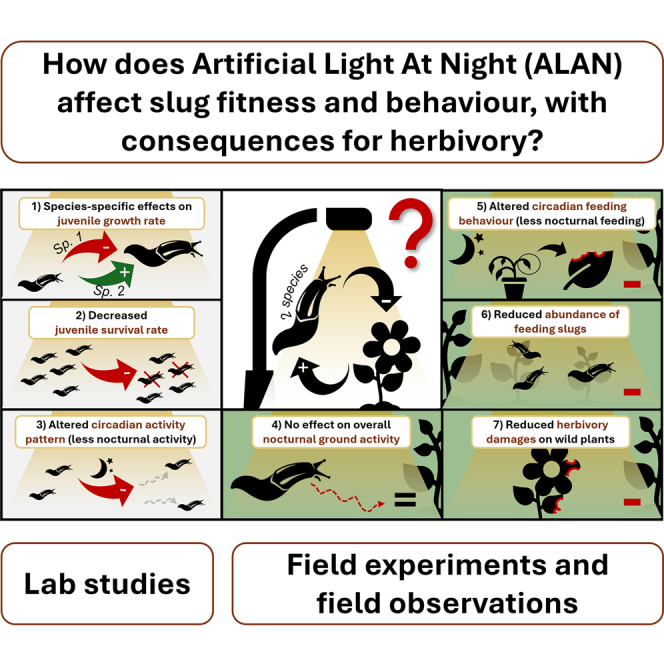

## Introduction

Artificial light at night (ALAN) is rapidly expanding worldwide,^1^^,^^2^^,^^3^^,^^4^ and evidence of its profound effects on biodiversity are increasingly numerous.^5^^,^^6^^,^^7^^,^^8^ A growing body of research demonstrates that ALAN influences biodiversity across ecological scales, altering not only ecosystem functioning,^9^^,^^10^ community composition,^11^ and populations dynamics^12^^,^^13^^,^^14^ but also species abundance^15^^,^^16^ and their interactions. However, although much effort is still devoted to studying behavioral and physiological responses to ALAN, its effects on ecosystem functions remain relatively underexplored. Recent research has explored how ALAN impacts key ecological processes such as predation,^17^^,^^18^^,^^19^ decomposition,^20^^,^^21^^,^^22^ and pollination,^23^^,^^24^^,^^25^ yet there is a particular “blind spot” regarding its impact on herbivory.

So far, both an increase^26^^,^^27^ and a decrease^28^ in herbivory due to ALAN have been observed. Furthermore, it remains unclear how ALAN influences the palatability of plants to herbivores, as studies have shown mixed effects in both directions.^12^^,^^13^^,^^14^ Most research on this topic does not rely on direct observations, and the few experiments conducted in controlled environments have tested the mechanisms linking ALAN to herbivory, primarily using lepidopterans larvae,^14^^,^^27^ orthopterans,^29^ and freshwater snails.^13^^,^^30^ To the best of our knowledge, the effect of ALAN on feeding activity of gastropods in terrestrial ecosystems has not yet been investigated.

The little attention that slugs and snails have received in terms of ecological impacts of ALAN is surprising because they are one of the most diverse groups of land animals (about 24,000 species) and occur in a wide range of habitats.^31^ Though they are mostly considered to be nocturnal, very little is known about their circadian feeding habits (e.g., Grimm and Schaumberger^32^). They not only play a key role in ecosystem functioning by promoting decomposition, nutrient cycling, and soil-building processes^33^^,^^34^^,^^35^ but also serve as food sources and provide essential nutrients to higher trophic levels.^36^^,^^37^^,^^38^^,^^39^ In Europe, 21.8% of the 2,469 native species of terrestrial molluscs are considered threatened (i.e., critically endangered, endangered, or vulnerable), with many more listed as at risk regionally and nationally.^40^ At the same time, several mollusc species are significant agricultural pests, causing crop damage with potentially severe economic loss.^41^ Understanding the direct and indirect effects of ALAN on slug-mediated herbivory is therefore crucial, particularly in areas where urban environments intersect with productive, minimally illuminated agricultural landscapes.

In this study, we examined the effects of ALAN on two widely distributed and destructive slug species, *Arion lusitanicus* and *Deroceras reticulatum*, which are common pests in temperate regions.^42^ We combined laboratory experiments with field observations to address the following overarching question: How does ALAN influence slug fitness and activity, and what are the implications for herbivory? To effectively guide this research, we addressed seven specific questions: (1) Does ALAN increase the juvenile growth rate (considered as a critical period for determining their long-term survival and usually taken for measuring fitness in invertebrates^43^) of the two species? (2) Does ALAN increase the juvenile survival rate of the two slug species? (3) Does ALAN affect circadian activity pattern in slugs? (4) Does ALAN increase overall nocturnal ground activity? (5) Does ALAN affect circadian feeding behavior of slugs? (6) Does ALAN increase abundance of slugs feeding on plants (further referred to as abundance of feeding slugs)? (7) Does ALAN lead to greater herbivory damage on wild plants? Questions 1 to 3 were explored in laboratory settings using bred specimens, whereas questions 4 to 7 were addressed through field experiments quantifying slug activity and feeding damage. Given that *A. lusitanicus* and *D. reticulatum* are especially active shortly after sunset,^32^^,^^44^ and considering that ALAN tends to reduce darkness to a level of light close to sunrise or sunset, we hypothesized that both species would extend their activity periods. This extended activity in response to ALAN has been reported in some other organisms that are active at dawn and dusk, such as songbirds.^45^^,^^46^^,^^47^ Therefore, we predicted that slugs would generally benefit from the extended period of activity enabled by ALAN. Specifically, we anticipated that ALAN would speed up slug juvenile growth rate, increase survival, shift circadian activity toward more nocturnal activity (question 1 to 3), enhance nocturnal ground and feeding activity, increase the abundance of feeding slugs, and amplify herbivory damage on wild plants (question 4–7).

## Results

### Juvenile growth rate

**Figure 1:**
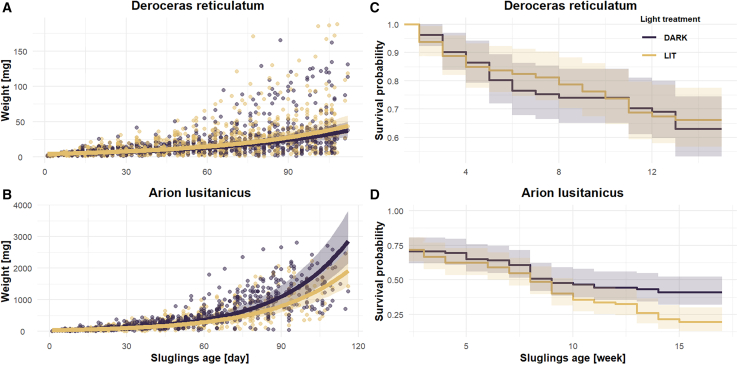
Effects of ALAN on juvenile growth and survival rate (A and B) (A) Growth rate of *Deroceras reticulatum* (*n* = 200) and (B) *Arion lusitanicus* (*n* = 209). The juvenile growth rates were analyzed using log-transformed mass to meet model assumptions. The data have been back-transformed in this figure to the original scale for visualization. (C and D) (C) Juvenile survival rate of *D. reticulatum* and (D) *A. lusitanicus*. Shaded area represent the 95% confidence intervals in all panels.

To investigate the impact of ALAN on juvenile growth, we reared juveniles *A. lusitanicus* (*n* = 209) and *D. reticulatum* (*n* = 200) under controlled laboratory conditions, with both natural and artificial light, and measured their weekly weight gain (question 1) and survival rate (question 2) over 100 days. ALAN had a species-specific effect on the growth curves of captive slugs, reducing the daily weight gain of *A. lusitanicus* (*p* < 0.001, β = −1.003, 95% confidence interval [CI] = −1.005 to −1.001) while increasing the daily weight gain of *D. reticulatum* (*p* = 0.022; β = 1.0015, CI = 1.000–1.001; Figures 1A and 1B; Table S2). Predicted values and confidence intervals are shown on the original response scale after back-transformation. After 90 days, *A. lusitanicus* grown under ALAN treatment were 24.76% lighter (based on the slug-averaged model) compared to individual reared under control conditions (Figure 1A; Table S2). In contrast, *D. reticulatum* exposed to ALAN were 11.9% heavier compared to those reared under natural light conditions (Figure 1B; Table S2).

### Juvenile survival rate

Additionally, ALAN influenced the survival rate of one of the two species investigated under captive conditions (question 2). Specifically, artificially illuminated *A. lusitanicus* had a 1.5-fold increased likelihood of mortality (*p* = 0.027, CI = 1.05–2.11) compared to non-illuminated *A. lusitanicus* (Figures 1C and 1D; Table S3). We found no significant effect of ALAN on the survival rate of *D. reticulatum* (*p* = 0.7; Figures 1C and 1D; Table S3).

### Circadian activity pattern

**Figure 2:**
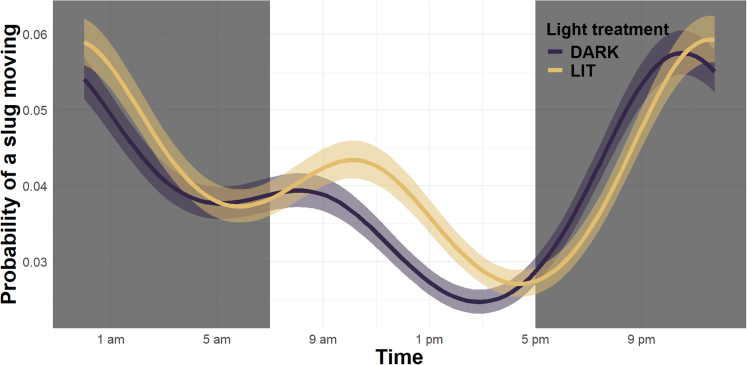
Effect of ALAN on the circadian activity pattern of captive *Arion lusitanicus* (*n* = 116) over a 24-h cycle The *x* axis represents the 24-h cycle, while the *y* axis shows the predicted probability of a slug moving between two images taken 15 min apart (based on the week-averaged model). Shaded areas around each line indicate the 95% confidence intervals, and the gray polygons represent the periods when the lamps were switched on. The sinusoidal model captures the rhythmic behavior of slugs and the differences between the two groups, along with their confidence intervals, highlighting the potential impact of ALAN on circadian activity.

To investigate the effect of ALAN on the circadian activity patterns of slugs (question 3), we used cameras to monitor the movements of a subset of *A. lusitanicus* reared in captivity under artificial light and dark control conditions. ALAN exerted a time-specific effect on the circadian activity of captive individuals, significantly reducing their mobility under ALAN treatment compared to controls during the early part of the night (6:00 p.m.–9:00 p.m.; no overlap of the confidence intervals). Conversely, they exhibited increased activity during the middle of the day (10:00 a.m.–3:00 p.m.; no overlap of the confidence intervals; Figure 2). Throughout the experiment, slugs exposed to ALAN were more active (*p* = 0.003; log odds ratio [OR] = 0.05; CI = 0.017–0.083; Table S4). A complementary analysis (2-h blocks model; see the statistical analysis section for details) corroborated the nighttime decrease in activity between 7:00 and 9:00 p.m., consistent with the above presented sinusoidal model. However, daytime activity patterns differed: the sinusoidal model indicates increased activity in the afternoon (Figure 2), whereas the block model revealed a significant increase in activity in the early morning (7:00–9:00 a.m.; Figure S1; Table S5).

### Nocturnal ground activity

To test the effect of ALAN on the nocturnal ground activity of slugs in the wild (question 4), we monitored slugs activity in illuminated and control wildflower stripes (WFS) using plastic track plates covered with graphite. The results showed that ALAN did not affect the nocturnal ground activity of wild slugs (probability of a plate to be visited by slugs: *p* = 0.132; surface of positive plates covered by slug tracks: *p* = 0.127). There was also no effect of temperature and no significant interaction between light treatment and temperature on slug activity on track plates. The nocturnal ground activity of wild slugs was positively correlated with slug abundance in the field (probability of a plate to be visited by slugs: *p* < 0.001; surface of positive plates covered by slug tracks: *p* = 0.007; Table S6).

### Circadian feeding behavior

**Figure 3:**
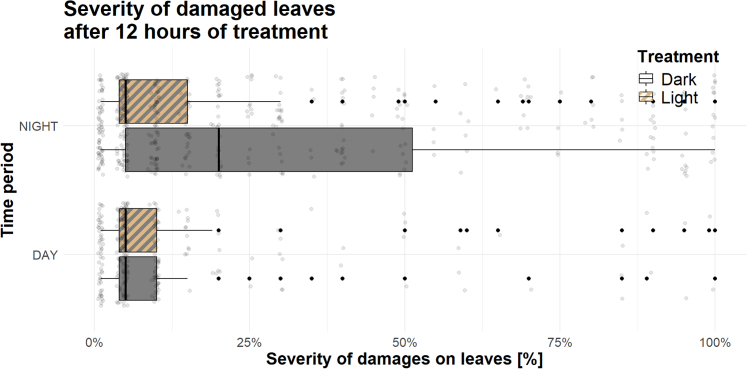
Effects of ALAN on slug circadian feeding behavior (severity of damaged leaves) on potted *Centaurea jacea* (*n* = 216)

To assess the effect of ALAN on the circadian feeding behavior of wild slugs (question 5), we exposed potted *Centaurea jacea* (*n* = 216) in illuminated and control WFS sites for 24 h, measuring feeding damage caused by slugs during both day and night. Wild slugs mainly fed on potted *C. jacea* during the night, and nocturnal herbivory damage was significantly reduced under ALAN conditions (proportion of damaged leaves: *p* = 0.016, OR = −2.12, CI = −4.06 to −1.15; severity of damage leaves: *p* = 0.024, β = −1.70, CI = −2.69 to −1.07; Figure 3). Temperature decreased the proportion of damaged leaves (*p* = 0.022) but had no consequences on the severity of damage on eaten leaves (*p* = 0.4; Table S7).

### Abundance of feeding slugs

**Figure 4:**
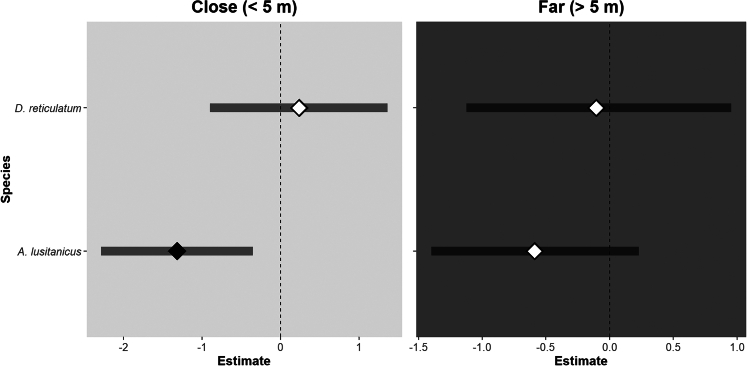
Effect of ALAN on abundance of feeding slugs (*n* = 416) Square-root-transformed species-specific estimated effects (median ±95% confidence interval) of the light treatment on total number of observed feeding slugs when only including individuals caught (left) along plots located close to the lamp (plots’ center located at less than 5 m) and (right) along plots located far from the lamp (plots’ center located at more than 5 m). See Figure S3 for more information regarding location of plots.

To address question 6 (effect of ALAN on the abundance of feeding slugs), we regularly counted and identified slugs feeding on wild plants in illuminated and control study sites, observing 416 slugs across 96 independent transects, with *A. lusitanicus* comprising the majority (75.7%) of the individuals. The results showed that ALAN not only significantly reduced the overall abundance of feeding slugs (*p* = 0.004, β = −1.96, CI = −5.29 to −0.20) but also revealed that the effect of ALAN was species-dependent. Specifically, the significant positive interaction between the light treatment and *D. reticulatum (p* = 0.029, β = 2.56, CI = 0.03 to 9.0) indicates that the negative effect of ALAN was weaker for *D. reticulatum* compared to *A. lusitanicus* (Table S8). Additionally, overall abundance of feeding slugs was not affected by the temperature (*p* = 0.2) or the size of the surveyed area (*p* = 0.2, Table S8). When analyzing separate models for the distance categories (FAR and CLOSE), ALAN reduced the number of *A. lusitanicus* feeding, and this effect was specifically pronounced in the model for plots located near the lamps (no overlap of the plotted CI the zero; Figure 4).

### Herbivory damage on wild plants

**Figure 5:**
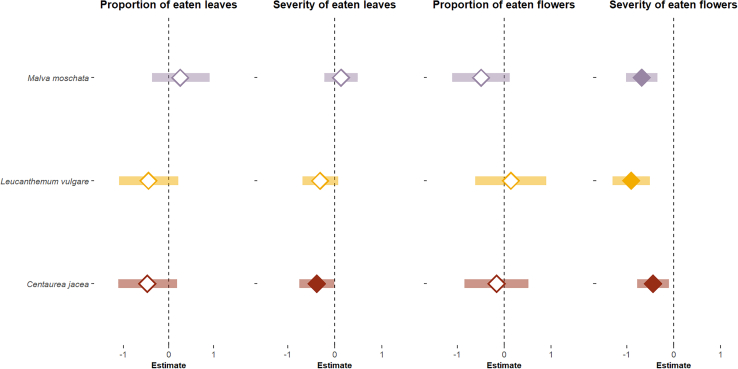
Effect of ALAN on herbivory damage on *Malva moschata* (*n* = 158), *Leucanthemum vulgare* (*n* = 172), and *Centaurea jacea* (*n* = 214) Log-transformed species-specific estimated effects (median +/− 95% confidence interval) of the light treatment on the number of eaten leaves, the severity of damaged leaves, the number of eaten flowers, and the severity of damaged flowers. Filled diamond (median) indicate that the CI do not overlap with the zeros.

To investigate the effect of ALAN on herbivory and florivory in the wild (question 7), we quantified feeding damage on wild *Malva moschata* (*n* = 158), *Leucanthemum vulgare* (*n* = 172), and *Centaurea jacea* (*n* = 214) in both illuminated and control WFS. Results revealed that ALAN significantly impacted the amount of feeding damage, though the effect varied across plant species. ALAN significantly reduced severity of florivory damage on eaten flowers (*p* = 0.033, β = −7.92, CI = −10.28 to −6.11), and we found no overlap with the zero and the CI for any of the three investigated plant species. To a lesser extent, ALAN reduced severity of foliar herbivory (*p* = 0.083, β = −1.47, CI = −2.29 to 1.05), and we found no overlap with the zero and the CI for *C. jacea*. We found no effect of ALAN on the proportion of damaged leaves (*p* = 0.231) or the proportion of damaged flowers (*p* = 0.698; Figure 5; Table S9).

## Discussion

Our study shows that ALAN can alter slug fitness and feeding activity, with consequences for the herbivory function they serve, under both controlled laboratory and field conditions. While ALAN tended to reduce activity and herbivory, these effects varied significantly by slug species and context, as discussed below.

### ALAN-induced decrease in slug herbivory

Contrary to our expectations, we observed reduced herbivory associated with ALAN in our field experiments. This was evident both when potted plants were exposed to natural herbivore communities for 24 h (question 5) and when feeding damage was measured on wild plants occurring in our study sites (question 7). We are confident that feeding damage on potted *C. jacea* was caused by terrestrial gastropods (question 5), as we only included damage with slug slime in our analysis. However, some feeding damage on wild flowers was likely caused by other herbivores (question 7). Given the surrounding agricultural fields and the dominance of slugs as herbivores, we believe most herbivory was caused by slugs. This is supported by data from a broader project (not shown here) showing that *A lusitanicus* and *D. reticulatum* accounted for 80% of plant-herbivore interactions at our study sites (see Figure S7 for details). Interestingly, our results contradict previous studies that reported increased herbivory under ALAN.^14^^,^^26^^,^^48^ However, unlike our findings, those studies primarily attributed the damage to insects. Since nocturnal insects are highly attracted to light source,^49^^,^^50^ plants growing near illuminated areas are visited more frequently by herbivorous insects, potentially explaining the increased damage observed in those cases. Moreover, our findings revealed that ALAN had a stronger effect on florivory than on foliar herbivory (Figure 5). This difference is likely due to the direct exposure of slugs to ALAN while feeding on flowers, often found at the top of plants. In contrast, foliar herbivory may be less affected, as foliage provides a protective cover. Although more speculative, another hypothesis is that ALAN influenced floral and foliar traits differently.^51^ Changes in plant morphology, nutritional dynamics, or defense mechanisms induced by ALAN may not be systemic but localized. For instance, some floral traits, such as petal palatability, could have been specifically altered by ALAN.^12^^,^^13^^,^^15^ However, regardless of whether or how ALAN affected plant traits, our experiment with potted plants suggests that ALAN clearly impacted slug behavior. Given that the *C. jacea* pots were exposed to ALAN for only one night (question 5), it is unlikely that traits mediating plant-herbivore interactions—such as leaf toughness and amount of phenolic compounds,^48^ C:N ratio,^12^ and plant biomass^52^—were sufficiently altered by ALAN within this short time frame. In summary, we propose that in a habitat where slugs are the dominant herbivores, plants—particularly their inflorescences—are less likely to be consumed when growing near light sources, as slugs tend to avoid areas with direct nocturnal illumination.

### Gradual impact of ALAN on slug activity

Consistent with our findings on reduced herbivory in the field (questions 5 and 7), the number of slugs feeding on plants along transects was also reduced by ALAN (question 6). This reduction was primarily driven by *A. lusitanicus* (accounting for 72.5% of the slugs caught), whereas the abundance of *D. reticulatum* feeding on plants was not significantly affected by ALAN. We observed that the abundance of *A. lusitanicus* feeding on plant material was particularly reduced in plots located close to the lamps (Figure 4), where nocturnal light intensity was relatively high, with an average light intensity of 29.0 ± 1.7 lx (mean ± SD), based on measurements taken at each corner of plots 01 and 04 in 2023 (Figure S4). In contrast, the effect was more moderate in plots farther from the lamps, where nocturnal light intensity was lower (9.26 ± 3.0 lx). These results suggest that *A. lusitanicus* tends to avoid strongly illuminated areas when feeding. Nevertheless, track plates (question 4) revealed no difference in slug visitation between dark and illuminated sites, indicating that ALAN did not affect nocturnal ground activity. One possible explanation, which in line with the observed difference between floral and foliar herbivory (question 7), is that slugs activity at ground level was not impacted by ALAN, since vegetation sheltered slugs from nocturnal light, allowing slugs to move beneath the plant foliage without being exposed. This might also explain the contrasting findings of van Grunsven et al. (2018)^53^ who found more slugs in pitfall traps placed under street lamps compared to dark locations. In their setup, the entire field was illuminated, attracting many insects. A portion of these insects likely died near the lamps, creating an additional food resource close to the ground, where slugs could feed while remaining hidden by the vegetation. Similar ALAN-mediated attraction of scavengers has been reported in carrion beetle *Silpha* sp.^54^ Overall, our results indicate that although ALAN significantly affects slugs foraging in areas with relatively high light intensity or on exposed plant parts, its impact appears minimal when light intensity is lower or when slugs can remain hidden from the light source, such as at ground level.

### ALAN-mediated disruption of slug circadian rhythms

Supporting our findings on the avoidance of light sources by wild slugs, captive *A. lusitanicus* under ALAN conditions were less active during the first few hours after sunset (Figure 2; Figure S1) compared to non-illuminated individuals (question 3). This suggests that, unlike some European songbird species, which have been shown to extend their activity in response to ALAN,^45^^,^^46^^,^^47^ slugs may instead reduce their activity and be significantly disturbed by it. This contrast likely reflects their differing activity patterns: in temperate regions, most slugs are nocturnal, whereas the songbirds are primarily active at dawn and dusk. This is consistent with the fact that environmental conditions during the night—such as higher humidity, lower temperatures, and the absence of direct sunlight—are ideal for terrestrial gastropods. Slugs also have sophisticated sensory systems that detect environmental brightness and trigger defense mechanisms in response to predator-cast shadows.^55^ Thus, it is likely that they also use light cues to align their circadian activity with the natural 24-h day-night cycle, although direct evidence is still lacking.^56^ As a result, ALAN may interfere with their perception of day and nights, disrupting their circadian rhythms and contributing to the reduced nocturnal activity we observed. Another possible assumption, which requires further testing, is that ALAN reduced the slugs’ nocturnal activity by increasing their perception of predation risk under illuminated conditions. As some predators’ ability to hunt increases with ALAN,^18^^,^^57^ slugs may have preferred to stay in safe place during the night instead of being active. Indeed, ALAN-induced predator avoidance at night has been found in other preys species,^58^^,^^59^ including not only invertebrates^60^^,^^61^ but also marine mollusc gastropods.^62^ The idea that slugs perceive a higher predation risk in illuminated sites is supported by their avoidance of feeding on exposed parts of plants (question 7), where they are more visible to predators, while being less affected by the light at ground level (question 5), where they remain sheltered by plant foliage. Interestingly, the sinusoidal model showed that ALAN-treated *A. lusitanicus* were more active between 10:00 a.m. and 3:00 p.m. compared to non-illuminated individuals (Figure 2), whereas the complementary 2-h blocks approach highlighted increased activity earlier in the morning (Figure S1). This discrepancy suggests that daytime activity patterns under ALAN are not well defined and may reflect model-specific sensitivities, potentially due to overfitting in the more flexible sinusoidal model. An explanation for a potential increased diurnal activity (either in the morning or afternoon) might be that the slugs increased their foraging activity during the day to compensate the time spent in safe place during the night. However, we believe that this increase in activity would not occur under real-life conditions. In our experiments, the slugs were provided with optimal temperatures and humidity levels, allowing them to move freely within the plastic boxes, even in broad daylight. In contrast, under natural conditions—particularly in summer—slugs are unlikely to expose themselves to direct sunlight while foraging. In sum, we conclude that ALAN disrupts the circadian rhythms of *A. lusitanicus*, reducing their nocturnal activity.

### Species-specific effects of ALAN on slug fitness parameters

Our findings of reduced nocturnal activity in *A. lusitanicus*, both in the field and under laboratory conditions, are further supported by decreased weight gain (question 1) and lower survival rate (question 2) observed in illuminated captive individuals (Figure 1). Although ALAN appeared to increase diurnal activity in the lab, illuminated *A. lusitanicus* gained less weight than non-illuminated individuals, suggesting that overall food intake did not increase. This ALAN-induced change in activity was likely suboptimal, contributing to both reduced growth—possibly due to increased energy expenditure from higher diurnal activity—and lower survival. Interestingly, we found the opposite effects of ALAN on *D. reticulatum* growth and no significant effects of ALAN on survival rate of the nightly illuminated individuals. Additionally, ALAN had only a marginal impact on the number of wild *D. reticulatum* feeding in the field (question 6). One possible explanation is that *D. reticulatum* is better adapted to diurnal abiotic conditions and exhibits greater flexibility in its temporal niche. Compared to *A. lusitanicus*, *D. reticulatum* is significantly lighter (the average weight of 100-day-old *D. reticulatum* is more than 10 times lower than a 100-day-old *A. lusitanicus*). This smaller body size likely allows *D. reticulatum* to take advantage of microstructures in their environment. These microstructures may create suitable microclimatic conditions that help mitigate the effects of heat from solar radiation, thereby enhancing their ability to withstand higher temperatures. They may have even benefited from ALAN by extending their feeding time (as hypothesized), explaining why ALAN-treated *D. reticulatum* gained more weight compared to non-illuminated slugs. However, this benefit seems to be limited, as we only found a marginal weight increase (+11.9% after 90 days) and no difference in survival rate between the two groups. Interestingly, opposite effects of light exposure on weight gain and growth curves were also found in other terrestrial slug species. For example, Hommay et al. (2001)^63^ found that *Limax valentianus* grew more slowly and weighed less under extended photoperiods than under natural conditions, whereas Udaka et al. (2008)^64^ reported that *Limax maximus* grew faster and were heavier with longer light exposure. However, instead of testing continuous nocturnal illumination, these studies examined only the effects of extended photoperiod (short-day [SD]: 12 h light and 12 h darkness vs. long-day [LD]: 16 h light and 8 h darkness) on captive slugs. In sum, we conclude that ALAN can alter the circadian activity pattern of terrestrial slugs with consequences for their fitness and survival. However, the effects are species-specific and appear to depend on the species’ preferred temporal niche.

Artificial light at night continues to increase worldwide,^4^ and it is increasingly being singled out for its wide-ranging effects on different ecosystem processes, communities, and groups of organisms.^8^ The results of the present study indicate that terrestrial gastropods have to be added to the already very long list of organisms impacted by ALAN, not only because we found that ALAN directly altered slug development growth and survival rate but also because we quantified that it indirectly reduces the proportion of suitable habitat where slugs can feed. Although slugs may be perceived as pests in the agricultural landscape, they play essential ecological roles in terrestrial ecosystems, notably by increasing decomposition of dead plant matter and promoting nutrient and carbon cycling.^35^ Slugs and snails are one of the most diverse group of land animals, occur in a large panel of habitat, and their impacts on ecosystems are numerous.^31^ Considerably more work will need to be done to determine how effects of ALAN on terrestrial gastropods could indirectly propagate on broader ecological scales and have consequences on species abundance, population dynamic, communities composition, and ecosystems functioning.

### Limitations of the study

Despite the contributions of our study, it should be noted that the light intensity simulated in the laboratory (24 lx), as well as in the field (see Figure S4), corresponds to values measured in the vicinity of commercial street lamps. Thus, the results of this study are applicable to situations where slug activity could be impacted by a comparable light source—such as directly at the roadside, in urban and peri-urban environments, or near illuminated infrastructure. This is consistent with the ability of terrestrial gastropods to inhabit a wide range of habitats,^31^ some of which may be directly exposed to ALAN. To better understand the broader-scale effects of ALAN on slug activity and fitness parameters, further research could investigate the impacts of ALAN at lower intensities, such as those caused by skyglow or exposure at greater distances from light sources. Additionally, only one of the most commonly used types of LED street lightings (4,000 K) was used for quantifying the impact of ALAN on herbivory. This, despite knowing that the activity of animals^65^^,^^66^^,^^67^ but also plant traits that mediate plant-herbivore interactions^48^^,^^51^ are responding differently when varying the ALAN spectrum. The study was, however, already quite complex and demanding, so that more combinations of spectra and intensities were not feasible. Also, the scope of the study was also not to test potential mitigation strategies but rather to test whether there is an effect at all. Thus, as part of follow-up studies, more spectra and intensities could be tested. Finally, our study was conducted in an intensively managed agricultural landscape where slugs dominated the community of herbivores. As insect herbivory showed various responses to ALAN,^12^^,^^13^^,^^52^ it is likely that the effects of ALAN on feeding damage rely on species composing the communities of herbivores.

## Resource availability

### Lead contact

Further information and requests for resources should be directly addressed to and will be fulfilled by the lead contact, Eva Knop (eva.knop@ieu.uzh.ch).

### Materials availability

This study did not generate new unique reagents.

### Data and code availability

• Original data have been deposited on Mendeley database and are publicly available as the date of publication. DOI is listed in the key resources table.
• All original codes have been deposited on GitHub and are publicly available as the date of the publication. Link to the codes is listed in the key resources table.
• Any additional information required to reanalyze the data reported in this paper is available from the lead contact upon request.

## Acknowledgments

We thank all who assisted with fieldwork (Martin Rais, Enzo Bertolo, Lara Plattner, Maxime Staelder, Katja Gisler, and Leonie Wüst) and the farmers who kindly provided access to field sites. This study was supported by the Swiss National Sciences Foundation (310030_197698).

## Author contributions

Conceptualization, V.G. and E.K.; methodology, V.G. and E.K.; formal analysis, V.G. and E.K.; investigation, V.G., J.C., and F.L.; writing—original draft, V.G.; writing—review & editing, V.G., F.L., J.C., and E.K.; visualization, V.G.; supervision, E.K.; funding acquisition, E.K.

## Declaration of interests

The authors declare no competing interests.

## STAR★Methods

### Key resources table

**Table:** 

REAGENT or RESOURCE	SOURCE	IDENTIFIER
**Deposited data**		

Original data deposited for this study	This study	Mendeley; https://data.mendeley.com/preview/z6xd3dnf7n?a=cac42ccd-8269-497f-9c1c-4ed87e2cc9be

**Experimental models: Organisms/strains**		

*Deroceras reticulatum* clutches	Zurich, Switzerland	N/A
*Arion lusitanicus* clutches	Zurich, Switzerland	N/A
*Deroceras reticulatum* feeding on plants	Seeland, Switzerland	N/A
*Arion lusitanicus* feeding on plants	Seeland, Switzerland	N/A

**Software and algorithms**		

Code for model building, evaluation, and plotting (R Code)	This study	Github; https://github.com/GrognuzV/Raw-data-for-ALAN-x-herbivory
R software v. 4.3.2	Core Team^68^	https://www.r-project.org
imagefx	Witsil^69^	https://doi.org/10.32614/CRAN.package.imagefx
lme4	Bates et al.^70^	https://doi.org/10.18637/jss.v067.i01
survival	Therneau^71^	https://cran.r-project.org/web/packages/survival/vignettes/survival.pdf

### Experimental model and study participant details

#### Lab experiments

Questions 1, 2 and 3 were investigated in the laboratory, using bred specimens of *Arion lusitanicus* and *Deroceras reticulatum*. In March 2023, we collected slug eggs from a total of 14 clutches of *D. reticulatum*, and in October 2023, from 18 of *A. lusitanicus* clutches, on agriculturally used fields near Zurich, Switzerland (47° 25′ 42.62″ N 8° 31′ 02.37″ E, UTM), and we immediately put them in glass containers. As soon as the slugs hatched, we recorded the hatching date and transferred them (*D. reticulatum*: n = 200 and *A. lusitanicus*: n = 209) individually into transparent plastic boxes (12 × 12 × 5 cm), at the bottom of which we prepared a 5 mm layer of plaster. Individuals of the different clutches were homogeneously distributed to six rooms of a building close to the sampling sites and placed next to north-facing windows in order to obtain natural light during daytime but at the same time not heating up due to direct sunshine. The average room temperature, recorded at each location using TOMST TMS-4 dataloggers (with one data point logged every five minutes), was 19.96 ± 0.79°C (standard deviation, SD). Three of these locations were artificially illuminated at night with a light bulb (osram E27, 4000 K, 4 W, 470 lm, 30 lux) hanging 1.5 m above the boxes. Using preprogrammed timers, the lights switched on and off synchronously with the weekly average civil sunset and sunrise time given for the location. Mean nocturnal light intensity measured on the top of the illuminated plastic boxes was 24.1 ± 1.24 lux and below 0.1 lux for the non-illuminated boxes (measured three hours after sunset on a moonless night, with testo 540 luxmeter, directly placed on the top of the boxes). We fed the slugs *ad libitum* with salad and we weekly shuffled the boxes within the three locations from the same light treatment.

#### Field experiments

Questions 4–7 were experimentally investigated in the Seeland region, Switzerland (Figure S2), in 2022 and in 2023. Due to high agricultural activity and a low human density, this region is characterized with a relatively low level of ALAN compared to the rest of the Swiss Lowland (21 mag/arcsec2, https://www.lightpollutionmap.info). We selected wild flower strips (WFS) that had never been directly illuminated, were located at least 500 m away from the closest source of artificial light at night (e.g., traffic street lamps) and were situated at more than 1000 m of any major source of light emissions (football field, airport, highly illuminated industrial building). Standardized seeds mixture from 34 indigenous plant species (listed in the Swiss federal ordinance for direct payments for biodiversity subsidies, Table S1) were sown in autumn in each study site by local farmers 3–5 years before we started the experiments (study sites area: 2’102–33’894 m2, mean = 12’981 ± 7’313 m2). Using a paired approach (2022: 6 pairs, 2023: 8 pairs), half of the study sites were illuminated at night from 15th of April to 15th of September, while control sites were kept dark. The paired sites were of similar age and located close to each other to ensure the most similar species communities, soil characteristic and forest cover in a 1 km radius around the lamps (distances between paired WFS: 661–5’272 m, mean = 2’333 ± 1’505 m). A LED streetlamp (SCHRÉDER: Ampera Midi 48 LED, color temperature: 4000 K, nominal LED flux: 6800 lm) was placed on top of a 6 m high telescopic mast (CLARK MASTS: CSQT6-4/HP, Figure S3C) in each of the illuminated sites, while dummy lamps (plastic box installed on top the same telescopic masts) were installed in dark control sites to ensure comparable conditions. Solar panels (SWISS VICTRON: Monocrystalline Solarpanel 175 W - 12 V) and portable batteries (ECOFLOW: RIVER 2 Pro 768 Wh) were used to provide electricity for the lamps (during long cloudy periods, the portable batteries were brought back to the research station and charged on the electricity network). Using timers, the lights switched on and off synchronously with the weekly average civil sunset and sunrise time given for the area. Light intensity followed a negative exponential curve as a function of the distance from the lamp starting from 48.8 ± 3.3 lx just under the lamp (<1 m) and falls below 1 lx after 12 ± 1.0 m in front the lamp, after 16.5 ± 1.0 m on the sides of the lamp, respectively (See Figure S4 for a detailed map of light intensity in the field and Figure S5 for information on the spectral range covered by the lighting system). In 2022, we delimited in each study site four parallel plots (1 × 25 m; tot = 48 plots), their center being located at 1.5, 3.5, 5.5 and 7.5 m in front of the lamps. In 2023, we delimited in all study sites 4 plots (2 × 4 m; tot = 64 plots), lining up in two rows of two plots located at 2 and 6 m in front of the lamps (Figure S3). In March 2023, we used a rototiller to plough the plots in which we sowed on the 19th of April a standardized seeds mixture (containing 22 annual indigenous plant species, presented in Table S1). We are confident that the different designs of the plots applied to the sites (linear plots from 2022 and tilled rectangle plots in 2023) did not affected our results (only questions 4 and 6 combine data from 2022 to 2023). As the plots from 2023 only represented a very small proportion (32 m2) of the entire wild flowers stripes (2’102–33’894 m2) we are confident that the ploughing treatment applied to the plots in March 2023 had no significant effects on the activity or communities of slugs living in the different study sites. In addition, the plant species that were sown in 2023 (Table S1) naturally occurred in the area and none of them are known to be especially attractive or repellent for slugs.

### Method details

#### Juvenile growth rate

To answer question 1*,* we weighed all juvenile slugs once a week at the nearest 0.1 mg using a precision balance (mettler AT261 Delta Range, accuracy of 0.005 mg) until they reached the age of 100 days.

#### Juvenile survival rate

To answer question 2*,* we weekly reported the number of dead individuals and replaced any deceased slugs with a new individual if death occurred within their 14 first days after hatching. Data for slugs that died within the first 14 days were excluded from subsequent analyses.

#### Circadian activity pattern

To answer question 3*,* we horizontally mounted two camera traps (reconyx, Hyperfire 2 professional small mammal camera) one meter above plastic boxes containing the slugs, in two distinct locations (one artificially illuminated and one dark control site). Each camera trap was set up in timelapse mode (one picture taken every 15 min, using an infrared flash every time the camera was triggered) and recorded the activity of randomly selected *A. lusitanicus* (n = 10–14 slugs per week and per treatment, mean = 11.6) during seven days and seven nights (in total, five independent one week-sessions from the 9^th^ of January to the 13^th^ of February 2024). A total of 116 *A. lusitanicus* (46.6% with light treatment) were surveyed during the entire experiment (the experiment was not reproduced for *D. reticulatum*). We analyzed all pictures (n = 6’554) using the R^68^ package imagefx.^69^ Based on optical flow algorithms,^72^ the package automatically counted number of slugs that moved between the picture taken at t_0_ and the following picture taken at t_+15 min_. We then tested for a time-specific effect of ALAN on the probability of a slug moving between two pictures taken 15 min apart (see the statistical analysis section for more details).

#### Nocturnal ground activity

To investigate question 4, we used polyvinyl chloride (PVC) plastic plates (16 × 24 cm) covered with a thin layer of graphite originally designed to record small mammal activity.^73^ As slugs crawl over the plates, they leave distinct traces on the graphite layer that are easily identified and quantified (Figure S6A). Track plates were placed in the different study sites between the 30^th^ of June and the 24^th^ of August 2022, and between the 11^th^ of May and the 24^th^ of August 2023. As slugs are rarely active during the day, track plates were only laid on dry nights, just before dusk, and were retrieved no later than one hour after dawn. We always sampled two study sites forming a pair (illuminated and control) on the same night (21 sampled nights, 2 to 5 pairs sampled per night, mean = 3.38, SD = 1.12), by laying plates at three fixed places located at 6.5 (±0.5 m) in front of the foot of the telescopic masts (see Figure S3 for a detailed plan of these locations). Track plates were always placed outside of plots. When recovering the plates, a photograph of each plate was taken. The pictures of the plates (n = 416) were then analyzed by a trained collaborator who visually estimated the proportion (in percentage) of plates covered by slug tracks.

#### Circadian feeding behavior

To answer question 5, we sowed *Centaurea jacea* seeds on the 18^th^ of April 2023 in germination trays with standardised soil in the greenhouses of the Agroscope Research Institute of Reckenholz and watered the trays at least twice a week. On the 23^rd^ of May, we transferred each seedling into individual pots and moved the pots to a site in our study area without artificial light sources within a radius of 100 m (46° 53′ 14.18″ N 7° 01′ 07.21″ E, UTM). The pots were regularly watered when needed. Between the 5^th^ of July and the 30^th^ of August, we randomly selected pots (none displaying inflorescences.) and exposed them to natural communities of herbivores in the different study sites (Figure S6B). As the pots were kept outside, some herbivory damage occasionally occurred before starting the experiment, though it was not often. Yet, to establish an accurate baseline for herbivory damage, we visually quantified the herbivory on the ten most eaten leaves just before bringing them to the field. Three pots were then placed in one of the two plots lined up in front of the lamp (P01 and P04) and three pots in one of the two plots lined up away from the lamp (P02 and P03, Figure S3; always six pots per study site and per sampling). Four study sites (two illuminated and two dark) were always sampled at the same time for 24 h (n = 24 pots per sampling for a total of 216 pots installed during 9 sampling rounds). We always controlled the pots twice (once in the morning, between 7:00 a.m. and 9:00 a.m. and once in the evening, between 6:00 p.m. and 8:00 p.m.) and visually quantified herbivory (in percentage) of the ten most eaten leaves during each control. Only herbivory damage made by terrestrial gastropods was considered (easily identified by traces of slime laid around the damage). The nocturnal damage was defined as the difference between the baseline measurements and the morning measurements, and the diurnal damage were measured as the difference between the late afternoon measurements and the morning measurements.

#### Abundance of feeding slugs

To answer question 6, we recorded the number of slugs observed while feeding on wild plants in all study sites on a regular basis (one sampling every three weeks, five samplings per year and per study sites) from the 20^th^ of May to the 1^st^ of September 2022 and 2023. We always sampled two sites forming a pair (one illuminated and one dark site) simultaneously, or at least within a time window of maximum two hours to minimize the measurement of effects on slug activity other than the light treatment. We recorded the number of feeding slugs within half of the plots from 2022 (we randomly selected two out of the four plots, one being located close to the lamp (T01 or T02) and one further away from the lamp (T03 or T04, Figure S2)) and in all the plots from 2023. All samplings started one hour after sunset and were finished before 2:00 a.m. We carefully examined all plants along the plots at a steady speed of 1 min observation time per m^2^ and recorded any observed slugs feeding on plant material (the time spent handling animals and to record the data being not counted as observation time). Every time a feeding slug was found, it was caught, transferred into an individual plastic vials, and the GPS location, collection time, and abiotic factors were recorded (rain, wind and temperature, provided in real time by meteoswiss). To avoid disturbance by artificial light, we used night-vision goggles to record feeding activity on dark sites (BIG25-CV, Vectronix). To test whether light differentially influenced the abundance of feeding slugs at various distances, the captured animals were categorized into two groups based on their location (slugs caught in T01, T02, P01, P04 being considered as CLOSE and the ones collected in the plots located further away from the lamp being considered as FAR; see Figure S3). At the end of the samplings, slugs were transferred into a freezer (-4°C) and were identified at the species level (only *A. lusitanicus* and *D. reticulatum* were observed during the entire project). This question was part of a broader project (not presented here) designed to assess the impact of ALAN on diurnal and nocturnal antagonistic interactions. To address question 6, only nocturnal plant-slug interactions were considered. An overview of all recorded diurnal and nocturnal interactions across different herbivore groups is provided in Figure S7.

#### Herbivory damage on wild plants

To answer question 7, we measured foliar herbivory and florivory damage on wild *Leucanthemum vulgare* (n = 172), *Centaurea jacea* (n = 214), and *Malva moschata* (n = 158), that grew in the different study sites. We measured from the 17^th^ of May to the 8^th^ of August 2023 individuals displaying at least one freshly opened inflorescence and being located close to the lamps (illuminated sites: 0.757–11.55 m, mean = 6.62 ± 3.52 m; dark sites: 0.58–54.05 m, mean = 8.19 ± 4.60 m). The plants were relatively evenly distributed within the treatments and the study sites (*L. vulgare*: from 4 lit to 3 dark sites, 50.6% of plants being illuminated; *C. jacea*: from 4 dark and 3 lit sites, 47.2% of plants being illuminated; *M. moschata*: from 6 lit to 6 dark sites, 50.4% of plants being illuminated). For each individual plant, we visually quantified (in percentage) the amount of foliar herbivory damage on the ten lowest leaves of the plant, as well as the amount of florivory on the five highest inflorescences.

### Quantification and statistical analysis

#### Juvenile growth rate

To answer question 1, we ran a linear mixed-effect models (LMM) using the function *lmer* from the lme4^70^ package in R. We separately analyzed the effects of ALAN on the two slug species, because weight of *A. lusitanicus* (mean weight after 100 days: 1351 ± 647.81 g) strongly differed from weight of *D. reticulatum* (mean weight after 100 days: 94.27 ± 38.00 g). As we expected an exponential growth of the individuals during their 100 first days of life, we used the log of the weight measurements as response variables and the age of the slugs (number of days since there are born) and the light treatment (two levels: dark and lit), as well as their interactions as exploratory variables. We included slug individual identities as random factor.

#### Juvenile survival rate

To answer question 2, we separately fitted for the two investigated species a regression model that have survival outcomes (Cox regression model^74^) using the *survdiff* function from the survival^71^ R-package. We included a two factors variable (0 = the slug survived the entire experiment, 1 = the slug died at some point of the experiment), a time variable (the age of the slugs for the ones who died, or the age of the slug at the end of the experiment for the ones that survived) to calculate a Ratio of Hazard (HR) between the light treatments. The HR is interpreted as the instantaneous rate of occurrence of the event of interest (here: death) in those who are still at risk for the event.

#### Circadian activity pattern

To answer question 3, we employed a Generalized Linear Mixed-effects Model (GLMM) to test for an effect of ALAN on the probability of a slug moving between two images taken 15 min apart. We used the cbind() function in R to combine the counts of moving and immobile slugs into a single response variable, and applied a binomial distribution as the underlying family. To account for the periodic nature of the 24-h cycle (i.e., 01:00 follows 12:00), we transformed the time variable (representing the 24 h of a 24-h period, where 01:00 h is recorded as 1 and 12:00 h as 24) into radians by dividing it by 24 ∗ 2 ∗ π. This radian-transformed time variable was then included in the model as a function of sine and cosine:

Probabilityofaslugmoving∼sin(time.radian)×light+cos(time.radian)×light+sin(2×time.radian)×light+cos(2×time.radian)×light

To account for variability across experimental sessions, we included experimental week as a random effect. Given the flexibility of harmonic models and the associated risk of overfitting, we also performed a complementary GLMM analysis. In this approach, observations were binned into 2-h intervals, and the average proportion of moving slugs per time block was modeled as a function of ALAN, using the same random effect structure as in the sinusoidal model.

#### Nocturnal ground activity

To answer question 4, we fitted GLMM with a binomial family with the presence of tracks (presence or absence) on the plates as response variable to test whether ALAN had a impact on the probability of a plate to be visited by slugs. We included light treatment (two levels: dark and lit), averaged temperature during the night and their interactions as exploratory variables. Since track plates record activity abundance, we included slug abundance per site as a covariate (specifically, the total number of slugs observed feeding on plant material throughout the season, as detailed in question 6) to more accurately assess the effect of ALAN on slug activity on the plates. Study site and sampling date were included as random factors. Additionally, to test whether the amount of activity on the positive plates (those with at least one percent of the plate covered by slug track) was impacted by ALAN, we fitted LMM using the square root (in order to fulfill the model assumptions) of the surface covered by slug tracks (in percentage) as response variable. We used the same exploratory variables and random factors as the ones used for the presence/absence analysis described above.

#### Circadian feeding behavior

To answer question 5, two parameters describing herbivory were analyzed, namely the proportion of eaten leaves and the severity of damaged leaves. In order to test whether ALAN treatment but also time period (day versus night) affected the proportion of eaten leaves, we fitted GLMM and we used the cbind() function in R to bind counts of damaged and undamaged leaves into a single response variable and used the binomial distribution as the underlying family. We included sampling period (two levels: day and night), light treatment (two levels: dark and lit), as well as their interaction and temperature (averaged values during the 12 past hours before each measurement) as exploratory variables. Study sites and sampling date were included as random factors. Then, in order to test whether ALAN treatment but also time period affected the severity of the damaged leaves, we fitted LMM using the log (in order to fulfill the model assumptions) of the herbivory severity estimated for each damaged leaf (i.e., the difference between the actual measurement and the previous measurement) as response variable. We used same exploratory variables and random factors as the ones used for the number of eaten leaves but added the ID of the pots as an extra random factor.

#### Abundance of feeding slugs

To answer question 6, we used GLMM with a negative binomial family using the *glmer.nb* command from the lme4 to test whether the presence of ALAN (but also the light intensity) impacted the abundance of feeding slugs recorded in our different study sites. We ran a model that included the square root (to fulfill model assumptions) of the number of observed feeding slugs as response variable and the light treatment (two levels: dark and lit), the distance between the lamp and the plots (two levels: close and far) and the slug species (two levels: D. reticulatum and A. lusitanicus) as well as their interactions (light ∗ distance ∗ species) as exploratory variables. In addition, we added the temperature and the size of surveyed the area (25 m^2^ in 2022 and 16 m^2^ in 2023, Figure S3) as exploratory variables and used a combination of the date and the pair of study sites as a random factor. Due to the extremely dry and hot summers, slugs were absent in a significant proportion of the sampling events (62.8% of samplings without slugs). For the analysis of the impact on ALAN on abundance of feeding slugs, we thus only kept samplings (i.e., two paired study sites) when at least one slug was found, resulting in 96 independent and paired sampling events.

#### Herbivory damage on wild plants

To answer question 7, two parameters describing herbivory and florivory were analyzed, namely the proportion of eaten leaves and flowers, as well as and the severity of damage on leaves and flowers (similarly to what we did in question 4). Thus, in order to test the effects of ALAN on the proportion of leaves/flowers presenting herbivory damage, we fitted GLMM with a binomial family and we used the cbind() function in R to bind counts of damaged and undamaged leaves/flowers into a single response variable and used the binomial distribution as the underlying family. We included the light treatment (two levels: dark and lit), the species (three levels: *M. moschata*, *L. vulgare* and *C. jacea*) and their interactions, as exploratory variables and the study site as random factor. Then, the effects of ALAN on damage severity (log-transformed percentage to meet model assumptions) on each damaged leave and flower (response variable) were analyzed using LMM, including light treatment (two levels: dark and lit), the species (three levels: *M. moschata*, *L. vulgare* and *C. jacea*) and their interactions as exploratory variables. We included study sites and plant identity as random factor.
