# Supplementary material for: Slugs hide in the dark: Artificial light at night alters fitness and activity of dominant herbivores with consequences for ecosystem functioning
Source: iScience. 2025 May 27;28(6):112770. doi: 10.1016/j.isci.2025.112770 (PMC12178802; doi:10.1016/j.isci.2025.112770)
Supplement: Document S1. Figures S1–S7 and Tables S1–S9 [file mmc1.pdf]

iScience, Volume *28*

Supplemental information

Slugs hide in the dark: Artificial light at night alters fitness and activity of dominant herbivores with consequences for ecosystem functioning

Vincent Grognuz, Flavienne Landolt, Julia Curty, and Eva Knop

1 SUPPLEMENTARY FIGURES

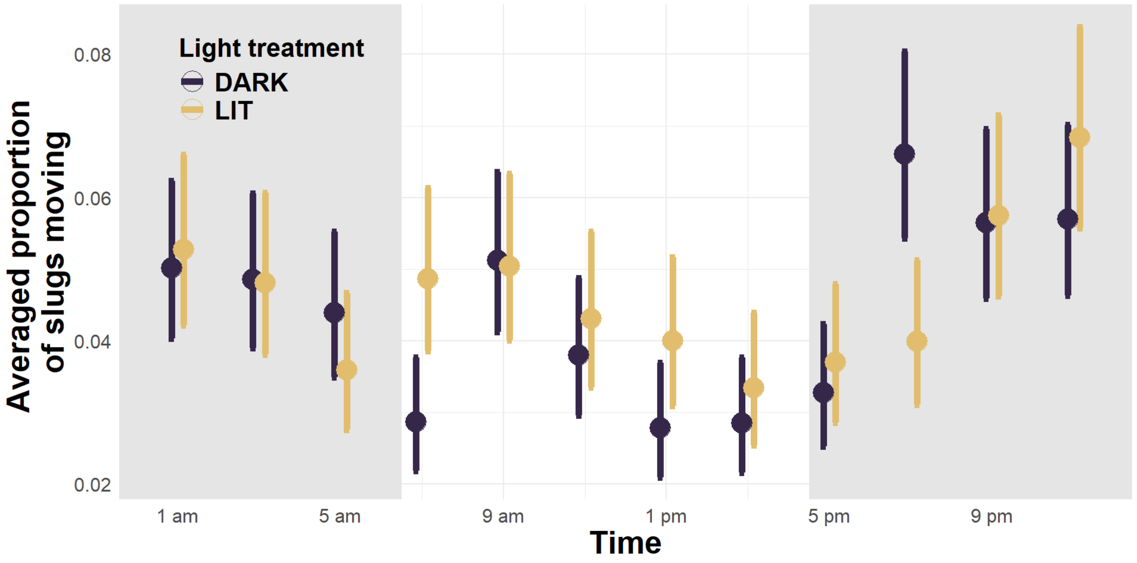

Figure S1: Effect of ALAN on the circadian activity pattern of captive *Arion lusitanicus* (*n* = 116) over a 24-hour cycle, divided into 12 blocks of two hours. The x-axis represents the 24-hour cycle divided into 2-hour blocks, and the y-axis shows the predicted average probability of a slug moving during each block. For example, the 9 am block corresponds to the average proportion of slugs that moved on pictures taken between 9:00 and 10:45 am. Dots indicate the mean predicted values, vertical bars represent 95% confidence intervals of the predicted average probability, and grey polygons mark the periods when lamps were switched on.

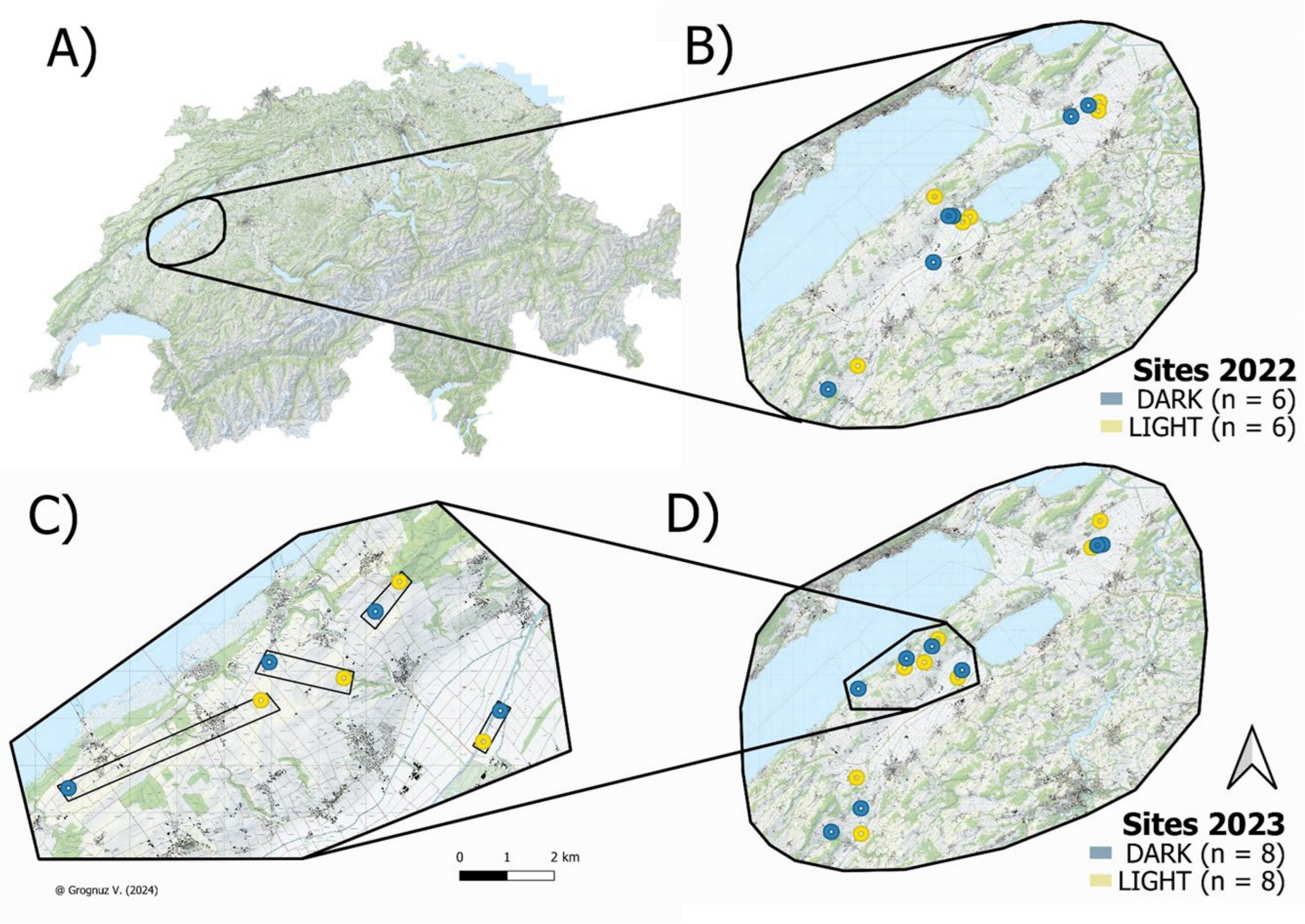

Figure S2: Study site locations. (**A**) Map of Switzerland; (**B**) study site locations from 2022; (**D**) study site locations from 2023; (**C**) close-up of the central study area showing paired study sites. Blue circles represent dark sites, yellow circles represent illuminated sites, and black rectangular polygons in (**C**) highlight four pairs of synchronously sampled sites.

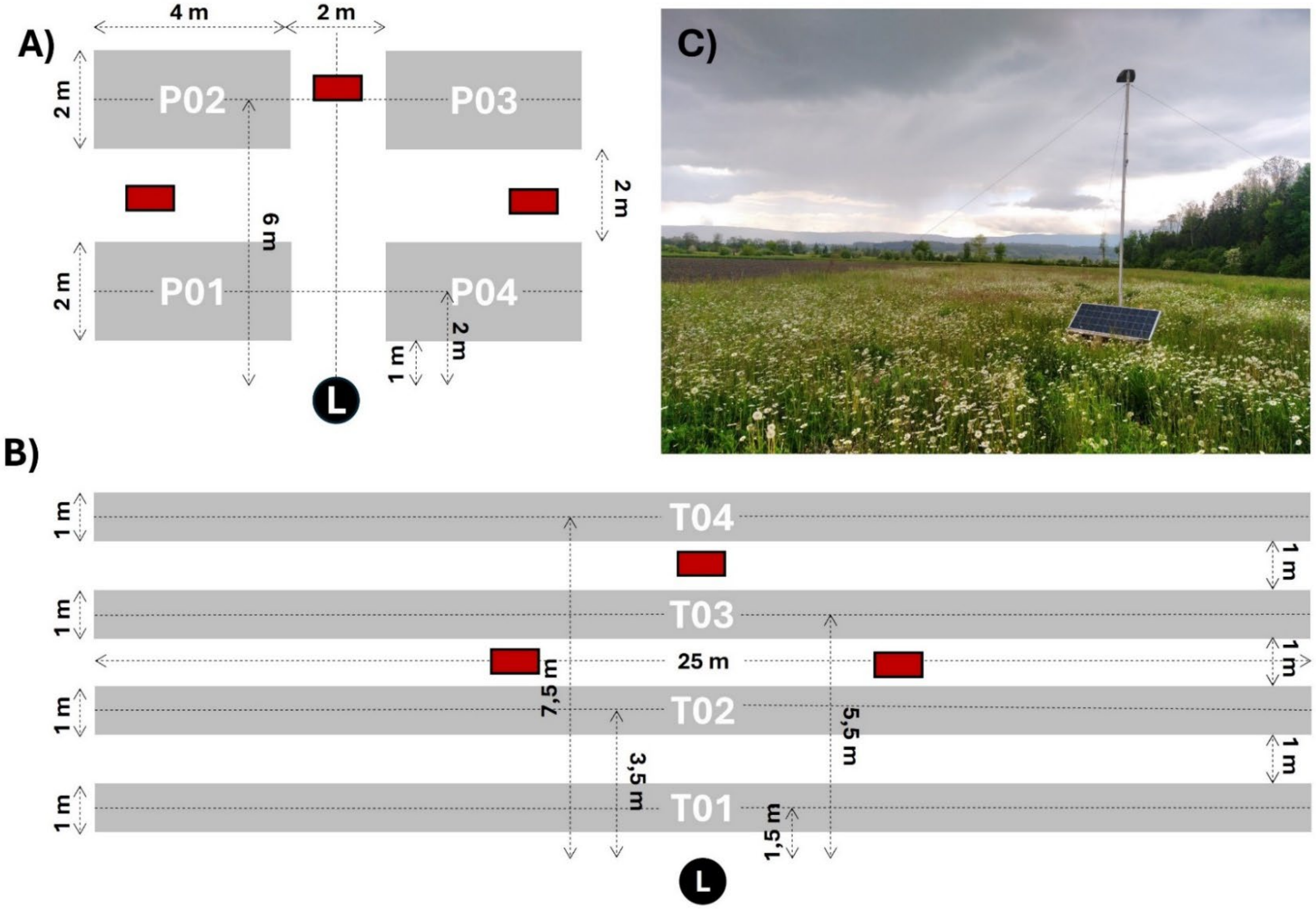

Figure S3: Disposition of the plots (T01 – T04 in 2022 and P01 – P04 in 2023), lamps (black point, included the letter "L") and track plates (red rectangles) in (**A**) 2023, (**B**) 2022 and (**C**) pictures of an illuminated study sites. The size of the track plates (red rectangles) do not respect the scale of the graph.

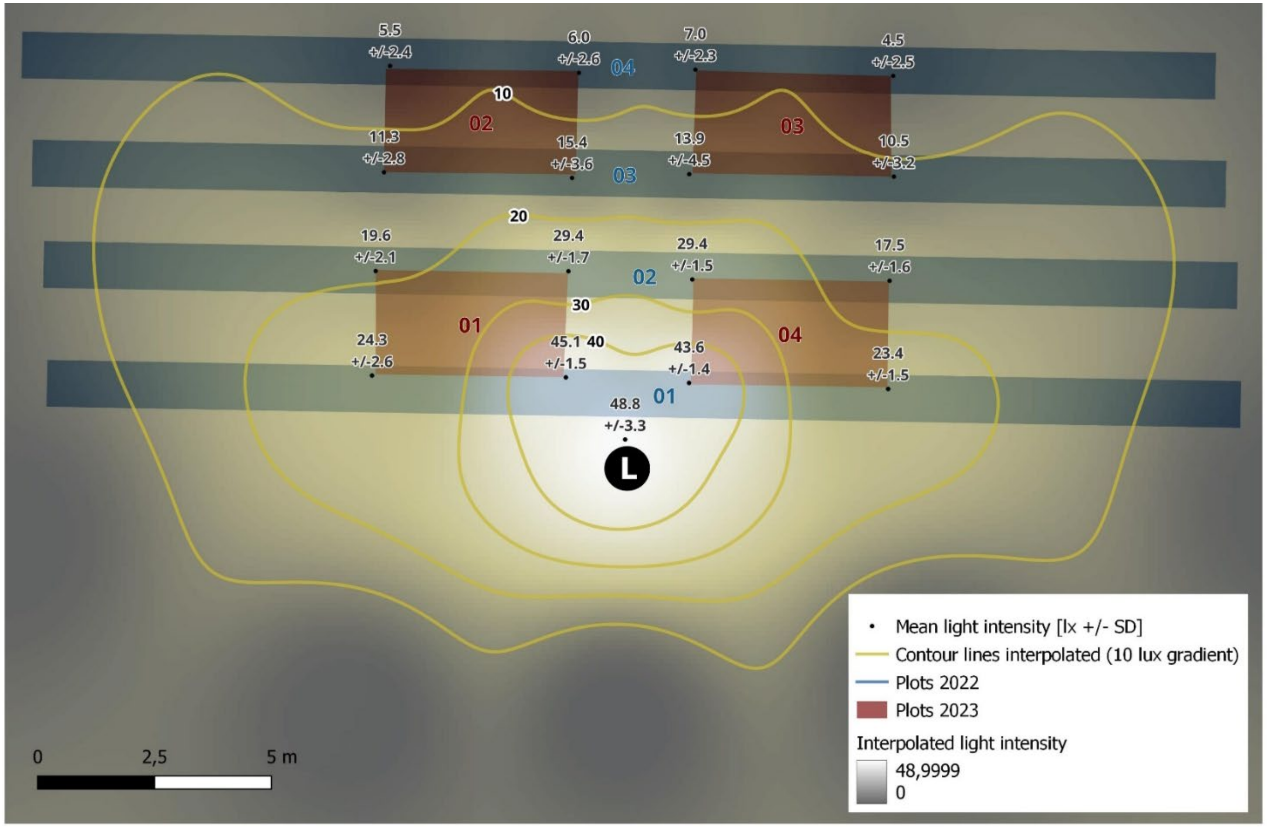

Figure S4: Light intensity over 2022 plots (blue) and 2023 plots (red). The black point labeled "L" represents the lamp location. Averaged light intensity values measured at fixed grid points in front of the lamp in the eight illuminated sites from 2023 were interpolated into a raster using the TRIANGULATED IRREGULAR NETWORK (TIN) INTERPOLATION tool in QGIS. Contour lines (yellow) were subsequently generated from this raster using the CONTOUR tool in QGIS[75]. The light measurements were performed at least two hours after sunset and in the absence of moon, using a TESTO 540 luxmeter, always keeping the sensor at 70 cm of height and pointing upward.

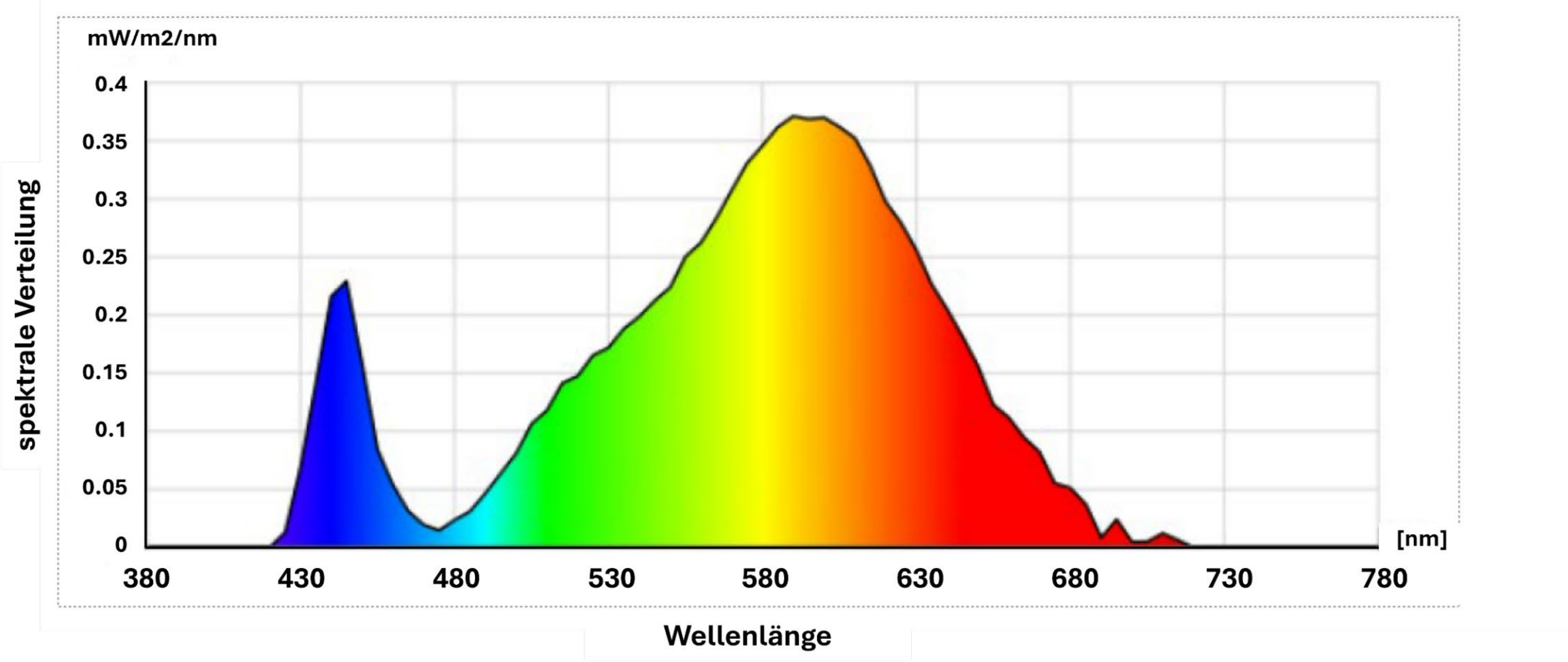

Figure S5: Spectral range covered by LED lamps (4000K) used for field experiment measured in illuminated field in 2023 using a spectrometer (GOSSEN: MAVOSPEC BASE)

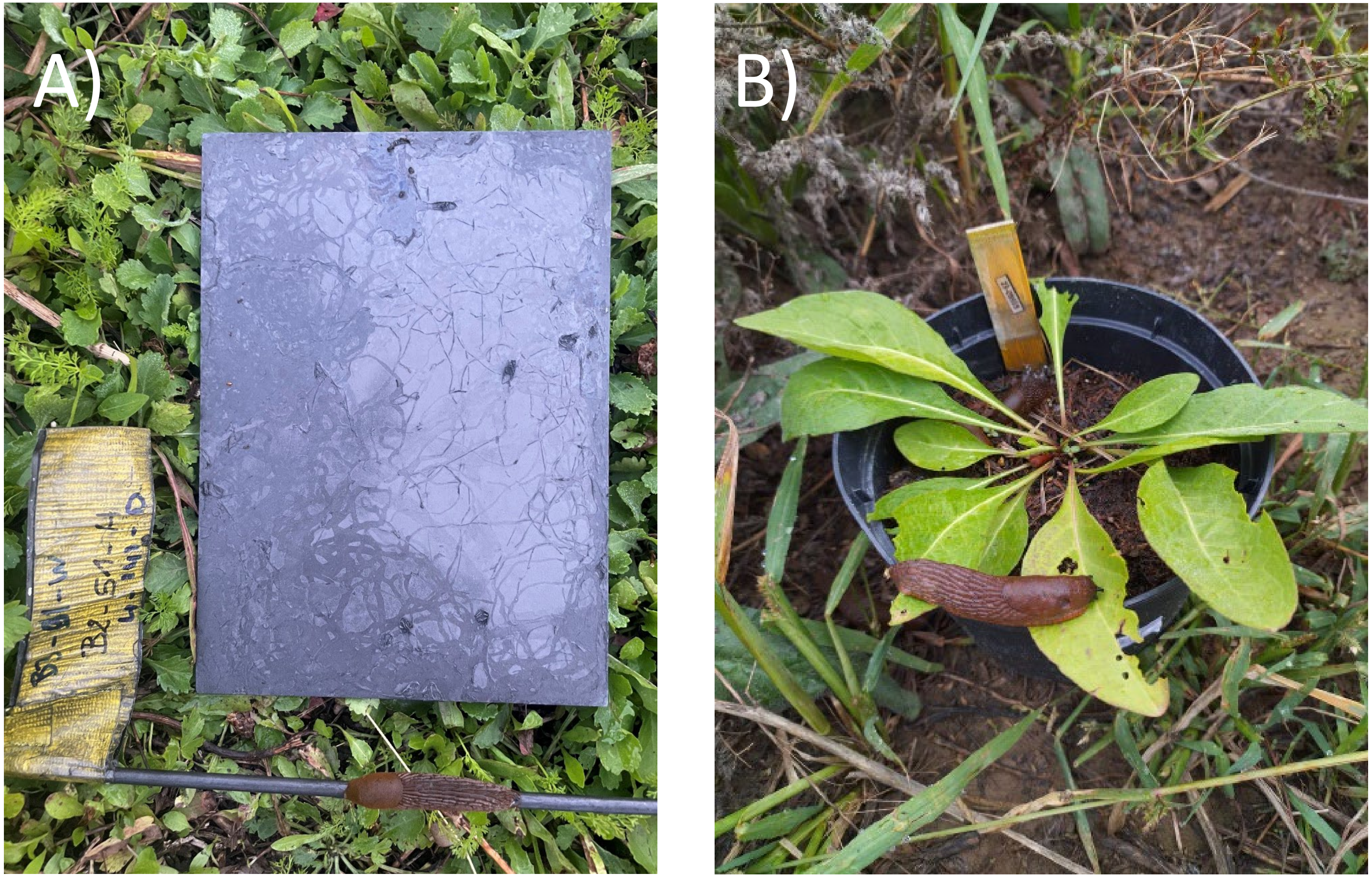

Figure S6: Methods used to quantify slug activity in the field (semi-natural experiment). An example of track plates, covered by slug slimes (A) and an example of potted *C. jacea* exposed for 24 hours in a study sites (B).

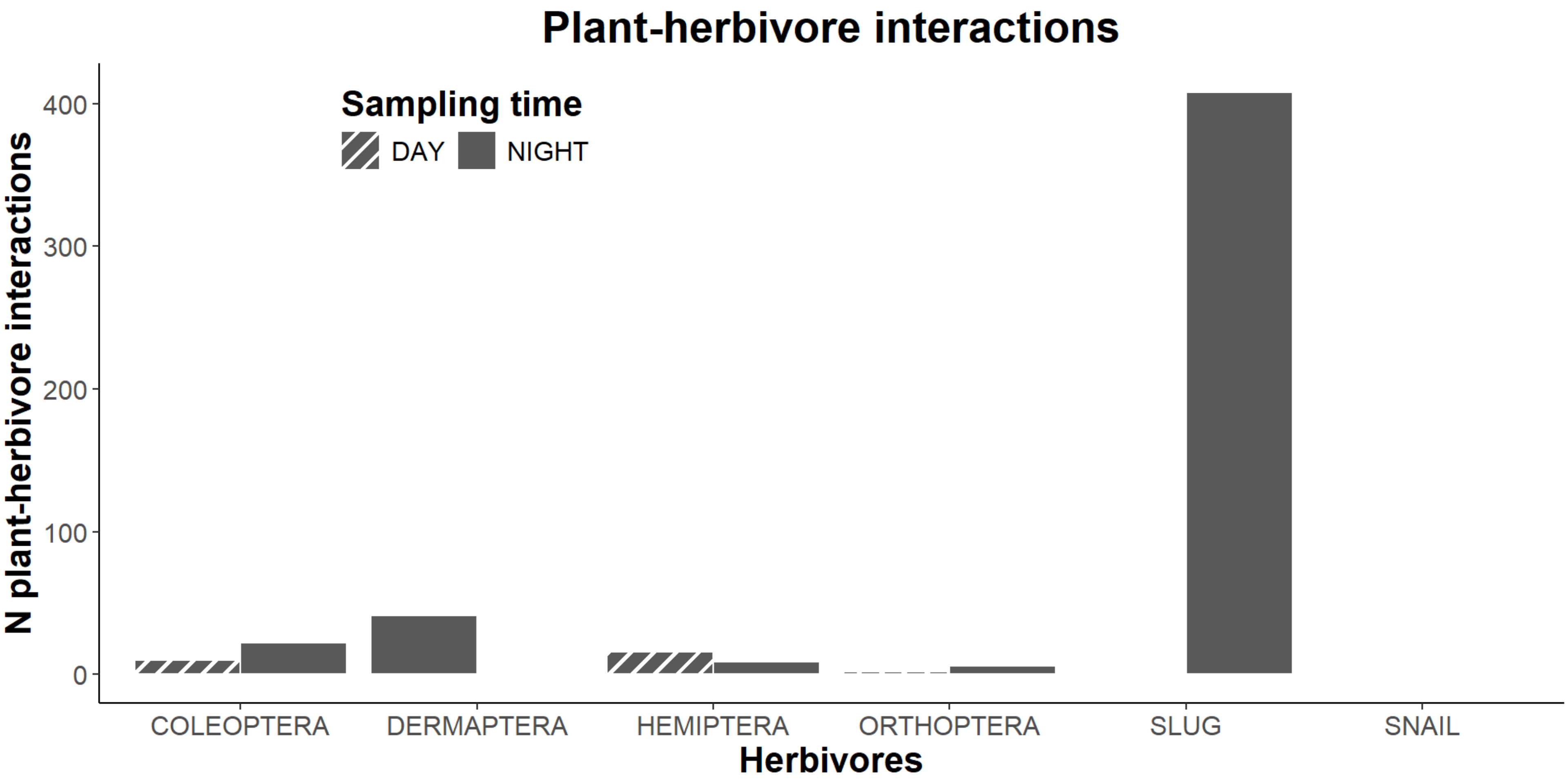

Figure S7: Number of diurnal and nocturnal plant-herbivore interactions recorded in 2022 and 2023 (n = 516) along transects across all study sites, grouped by herbivore type. For this study (question 6), only nocturnal plant-slug interactions were included.

SUPPLEMENTARY TABLES

Table S1: List of plants sown in Swiss conventional wild flowers stripes (based on Swiss federal ordinance for direct payments for biodiversity subsidies) and list of plants sown in tilled plots in 2023.

Family	Genus	Species	Sown by farmers in Swiss WFS	Sown by in plots
Asteraceae	*Achillea*	*millefolium*	x	
Caryophyllaceae	*Agrostemma*	*gigato*	x	x
Boraginaceae	*Anchusa*	*arvensis*	x	
Apiaceae	*Anethum*	*graveolens*		x
Asteraceae	*Anthemis*	*arvensis*		x
Asteraceae	*Anthemis*	*tinctoria*	x	x
Boraginaceae	*Buglossoides*	*arvensis*	x	
Brassicaceae	*Camelina*	*sativa*	x	
Asteraceae	*Centaurea*	*cyanus*	x	x
Asteraceae	*Centaurea*	*jacea*	x	x
Asteraceae	*Cichorium*	*intybus*	x	x
Lamiaceae	*Clinopodium*	*vulgare*		x
Ranunculaceae	*Consolida*	*regalis*	x	
Apiaceae	*Coriandrum*	*sativum*		x
Asteraceae	*Crepis*	*capillaris*		x
Apiaceae	*Daucus*	*carota*	x	
Caprifoliaceae	*Dipsacus*	*fullonum*	x	
Boraginaceae	*Echium*	*vulgare*	x	
Polygonaceae	*Fagopyrum*	*esculentum*	x	
Hypericaceae	*Hypericum*	*perforatum*	x	
Asteraceae	*Hypochaeris*	*radicata*		x
Campanulaceae	*Legousia*	*speculum-veneris*	x	x
Asteraceae	*Leucanthemum*	*vulgare*	x	
Fabaceae	*Lotus*	*corniculatus*		x
Malvaceae	*Malva*	*sylvestris*	x	
Malvaceae	*Malva*	*moschata*	x	x
Fabaceae	*Melilotus*	*albus*	x	
Fabaceae	*Onobrychis*	*viciifolia*	x	
Fabaceae	*Ononis*	*spinosa*		x
Lamiaceae	*Origanum*	*vulgare*	x	
Papaveraceae	*Papaver*	*dubium*	x	
Papaveraceae	*Papaver*	*rhoeas*	x	x
Apiaceae	*Pastinaca*	*sativa*	x	
Resedaceae	*Reseda*	*lutea*	x	
Lamiaceae	*Salvia*	*pratensis*		x
Caryophyllaceae	*Silene*	*latifolia*		x
Caryophyllaceae	*Silene*	*noctiflora*	x	
Brassicaceae	*Sinapis*	*arvensis*		x
Lamiaceae	*Stachys*	*annua*	x	x
Asteraceae	*Tanacetum*	*vulgare*	x	
Asteraceae	*Tragopogon*	*orientalis*	x	
Fabaceae	*Trifolium*	*incarnatum*		x
Fabaceae	*Trifolium*	*pratense*		x
Caryophyllaceae	*Vaccaria*	*pyramidata*	x	
Scrophulariaceae	*Verbascum*	*densiflorum*	x	
Scrophulariaceae	*Verbascum*	*lychnitis*	x	

Table S2: Results of LMM testing the effects of juvenile slug age, light treatment (no ALAN vs. ALAN) and their interactions on slugs weight, for *Deroceras reticulatum* (n = 200) and *Arion lusitanicus* (n = 209). Values are presented on the log-transformed scale.

**Characteristic**	**Deroceras reticulatum**				**Arion lusitanicus**			
	**Beta**	**SE**[1]	**95% CI**[1]	**p-value**[2]	**Beta**	**SE**[1]	**95% CI**[1]	**p-value**[2]
**(Intercept)**	1.227	0.076	1.0882 - 1.3920	**<0.001*** **	3.588	0.067	3.4693 - 3.7394	**<0.001*** **
**AGE**	0.019	0.000	0.0185 - 0.0207	**<0.001*** **	0.037	0.001	0.0355 - 0.0377	**<0.001*** **
**LIGHT**				0.9				>0.9
DARK	—	—	—		—	—	—	
LIT	-0.019	0.106	-0.2111 - 0.2198		0.001	0.093	-0.2267 - 0.1627	
**AGE * LIGHT**								
*AGE * LIT*	0.0015	0.0007	0.0002 - 0.0027	**0.022***	-0.0032	0.0009	-0.0049 - -0.0014	**<0.001*** **

[1] SE = Standard Error, CI = Confidence Interval

[2] *p<0.05; **p<0.01; ***p<0.001

Table S3: Results of LMM testing the effects light treatment (no ALAN vs. ALAN) on juvenile survival rate for *Deroceras reticulatum* (n = 200) and *Arion lusitanicus*.(n = 209).

**Characteristic**	**Deroceras reticulatum**			**Arion lusitanicus**		
	**HR**[1]	**CI**[1]	**p-value**[2]	**HR**[1]	**CI**[1]	**p-value**[2]
**LIGHT**						
DARK	—	—		—	—	
LIT	0.91	0.54 - 1.52□	0.7	1.49	1.05 - 2.11□	**0.027***

[1] HR = Hazard Ratio - CI = Confidence Interval

[2] *p<0.05; **p<0.01; ***p<0.001

Table S4: Results of GLMM (sinusoidal model) testing the effects of light treatment (no ALAN vs. ALAN) and time, as well as their interactions on the averaged circadian activity pattern of captive *Arion lusitanicus*.

**Characteristic**	**log(OR)**[1]	**SE**[1]	**95% CI**[1]	**p-value**[2]
**(Intercept)**	-3.245	0.09	-3.415, -3.076	**<0.001*** **
**LIGHT**				**0.003** **
DARK	—	—	—	
LIT	0.05	0.02	0.017, 0.083	
**sin(TIME.radian)**	0.065	0.02	0.033, 0.098	**<0.001*** **
**cos(TIME.radian)**	0.302	0.02	0.270, 0.334	**<0.001*** **
**sin(2 * TIME.radian)**	-0.188	0.02	-0.220, -0.156	**<0.001*** **
**cos(2 * TIME.radian)**	0.067	0.02	0.035, 0.098	**<0.001*** **
**sin(TIME.radian) * LIGHT.TREA**				0.050
*sin(TIME.radian) * LIT□*	0.047	0.02	0.000, 0.094	
**LIGHT.TREA * cos(TIME.radian)□**				**<0.001*** **
*LIT * cos(TIME.radian)□*	-0.108	0.02	-0.153, -0.063	
**LIGHT.TREA * sin(2 * TIME.radian)□**				**0.009** **
*LIT * sin(2 * TIME.radian)□*	0.061	0.02	0.015, 0.107	
**LIGHT.TREA * cos(2 * TIME.radian**				**<0.001*** **
*LIT * cos(2 * TIME.radian)*	0.12	0.02	0.074, 0.166	

1 OR = Odds Ratio, SE = Standard Error, CI = Confidence Interval

2 *p<0.05; **p<0.01; ***p<0.001

Table S5: Results of GLMM (2-hours blocks model) testing the effects of light treatment (no ALAN vs. ALAN) and time, as well as their interactions on the averaged circadian activity pattern of captive *Arion lusitanicus*.

**Characteristic**	**log(OR)**[1]	**SE**[1]	**95% CI**[1]	**p-value**[2]
**(Intercept)**	-3.008	0.13	-3.264, -2.753	**<0.001**
**LIGHT**				0.732
DARK	—	—	—	
LIT	0.05	0.15	-0.237, 0.337	
**TIME (2-hours block)**	0.065	0.02	0.033, 0.098	**<0.001***
01	—	—	—	
03	-0.032	0.15	-0.316, 0.253	
05	-0.133	0.15	-0.424, 0.159	
07	-0.559	0.17	-0.885, -0.232	
09	0.021	0.14	-0.260, 0.301	
11	-0.278	0.16	-0.585, 0.029	
13	-0.589	0.17	-0.925, -0.254	
15	-0.564	0.17	-0.895, -0.234	
17	-0.428	0.16	-0.742, -0.113	
19	0.275	0.14	0.009, 0.542	
21	0.118	0.14	-0.157, 0.393	
23	0.128	0.14	-0.147, 0.402	
**LIGHT * TIME (2-hours block)**	0.065	0.02	0.033, 0.098	**0.003**
*LIT * 03*	-0.062	0.21	-0.474, 0.351	
*LIT * 05*	-0.253	0.22	-0.687, 0.182	
*LIT * 07*	0.477	0.23	0.035, 0.920	
*LIT * 09*	-0.066	0.21	-0.474, 0.342	
*LIT * 11*	0.074	0.22	-0.364, 0.512	
*LIT * 13*	0.312	0.24	-0.151, 0.775	
*LIT * 15*	0.109	0.24	-0.359, 0.577	
*LIT * 17*	0.074	0.23	-0.375, 0.522	
*LIT * 19*	-0.554	0.21	-0.966, -0.143	
*LIT * 21*	-0.032	0.2	-0.430, 0.366	
*LIT * 23*	0.132	0.2	-0.259, 0.522	

[1] OR = Odds Ratio, SE = Standard Error, CI = Confidence Interval
[2] *p<0.05; **p<0.01; ***p<0.001

Table S6: Results of GLMM testing the effects of light treatment (no ALAN vs. ALAN), temperature, their interactions and slug abundance on two different metrics (probability of finding slug tracks on track plates (n = 416) and the proportion of positive plates covered by slug tracks) of slug nocturnal locomotor activity.

	**Probability of a plate to be visited by slugs**				**Surface of positive plates covered by slug tracks**			
**Characteristic**	**log(OR)**[1]	**SE**[1]	**95% CI**[1]	**p-value**[2]	**Beta**[1]	**SE**[1]	**95% CI**[1]	**p-value**[2]
**(Intercept)**	0.044	1.94	-3.766, 3.854	0.982	4.032	1.59	0.923, 7.141	**0.011***
**LIGHT**				0.132				0.127
DARK	—	—	—		—	—	—	
LIT	2.514	1.67	-0.761, 5.789		2.289	1.5	-0.651, 5.229	
**TEMP**	-0.075	0.12	-0.307, 0.156	0.523	-0.003	0.09	-0.180, 0.174	0.973
**SLUGS_N**	0.057	0.02	0.027, 0.086	**<0.001*****	0.032	0.01	0.009, 0.055	**0.007****
**LIGHT * TEMP**				0.538				0.142
*LIGHT * TEMP*	-0.06	0.1	-0.250, 0.131		-0.12	0.08	-0.287, 0.041	

[1] SE = Standard Error, CI =

[2] *p<0.05; **p<0.01; ***p<0.001

Table S7: Results of GLMM and LMM testing the effects of light treatment (no ALAN vs. ALAN), time period (day vs. night), their interactions as well as temperature on two different herbivory metrics (proportion of damage leaves and severity of damage on eaten leaves) on potted Centaurea jacea (n = 216).

	**Proportion of damaged leaves**				**Severity of damages on eaten leaves**			
**Characteristic**	**log(OR)**[1]	**SE**[1]	**95% CI**[1]	**p-value**[2]	**Beta**[1]	**SE**[1]	**95% CI**[1]	**p-value**[2]
**(Intercept)**	-0.36	1.38	-3.1 - 2.3	0.8	-2.3	0.561	-3.4 - -1.2	**<0.001*** **
**TIME PERIOD**				**0.09***				**<0.001*** **
DAY	—	—	—		—	—	—	
NIGHT	0.68	0.4	-0.11 - 1.5		0.78	0.168	0.46 - 1.1	
**LIGHT**				0.8				0.7
DARK	—	—	—		—	—	—	
LIT	-0.09	0.303	-0.69 - 0.50		-0.09	0.251	-0.58 - 0.40	
**TEMP**	-0.14	0.06	-0.25 - -0.02	**0.022***	-0.02	0.023	-0.07 - 0.02	0.4
**TIME PERIOD * LIGHT**				**0.016***				**0.024***
*NIGHT * LIT*	-0.75	0.311	-1.4 - -0.14		-0.53	0.234	-0.99 - -0.07	

[1] OR = Odds Ratio, SE = Standard Error, CI = Confidence Interval

[2] *p<0.05; **p<0.01; ***p<0.001

Table S8: Results of GLMM testing the effects of light treatment (no ALAN vs. ALAN), class of distance between the plots and the lamps (close vs. far), slug species (*Arion lusitanicus* and *Deroceras reticulatum*) their interactions as well as temperature and size of the surveyed areas on the abundance of feeding slugs (n = 416) observed along 96 independent samplings.

**Characteristic**	**Beta**	**SE**[1]	**95% CI**[1]	**p-value**[2]
**(Intercept)**	2.1	1.37	-0.56 - 4.8	0.12
**LIGHT**				**0.004****
DARK	—	—	—	
LIGHT	-1.4	0.48	-2.3 - -0.45	
**DISTANCE**				0.5
CLOSE	—	—	—	
FAR	-0.26	0.403	-1.1 - 0.53	
**SPECIES**				**0.002****
A. lusitanicus	—	—	—	
D. reticulatum	-1.5	0.49	-2.5 - -0.53	
**TEMP**	-0.05	0.042	-0.13 - 0.03	0.2
**AREA**	-0.03	0.036	-0.10 - 0.04	0.5
**LIGHT * DISTANCE**				0.2
*LIGHT * FAR*	0.77	0.641	-0.49 - 2.0	
**LIGHT * SPECIES**				**0.029****
*LIGHT * D. reticulatum*	1.6	0.717	0.16 - 3.0	
**DISTANCE * SPECIES**				0.6
*FAR * D. reticulatum*	0.35	0.681	-0.98 - 1.7	
**LIGHT * DISTANCE * SPECIES**				0.3
*LIGHT * FAR * D. reticulatum*	-1.1	0.998	-3.0 - 0.90	

1 SE = Standard Error, CI = Confidence Interval

2 *p<0.05; **p<0.01; ***p<0.001

Table S9: Results of linear mixed-effect model testing the effects of light treatment (no ALAN vs. ALAN), plant species (Centaurea jacea, n = 214; Leucanthemum vulgare, n = 172; Malva moschata, n = 158) as well as their interactions on two foliar herbivory metrics (proportion of damage leaves and severity of damage on eaten leaves) and two florivory metrics (proportion of damage flowers and severity of flowers on eaten leaves)

	**Foliar herbivory**								**Florivory**							
	Proportion of damaged leaves				Severity of damages on eaten leaves				Proportion of damaged flowers				Severity of damages on eaten flowers			
**Characteristic**	**log(OR)**[1]	**SE**[1]	**95% CI**[1]	**p-value**[2]	**Beta**[1]	**SE**[1]	**95% CI**[1]	**p-value**[2]	**log(OR)**[1]	**SE**[1]	**95% CI**[1]	**p-value**[2]	**Beta**[1]	**SE**[1]	**95% CI**[1]	**p-value**[2]
**(Intercept)**	1.346	0.28	0.805 - 1.887	**<0.001*** **	2.06	0.15	-2.36 - -1.76	**<0.001**	0.376	0.29	-0.186 - 0.939	0.189	-2.07	0.13	-2.325 - -1.807	**<0.001**
**LIGHT**				0.231				0.083				0.698				**0.033***
DARK	—	—	—		—	—	—		—	—	—		—	—	—	
LIT	-0.472	0.39	-1.245 - 0.301		-0.39	0.22	-0.83 - 0.05		-0.165	0.43	-0.999 - 0.669		-0.44	0.21	-0.840 - -0.036	
**SPECIES**				**<0.001*** **				**<0.001*** **				0.486				**<0.001*** **
Centaurea jacea	—	—	—		—	—	—		—	—	—		—	—	—	
Leucanthemum vulgare	-0.834	0.11	-1.047 - -0.622		-0.06	0.1	-0.257 - 0.133		-0.147	0.28	-0.695 - 0.401		-0.06	0.19	-0.441 - 0.314	
Malva moschata	-0.929	0.13	-1.185 - -0.674		-0.85	0.12	-1.094 - -0.612		0.199	0.26	-0.311 - 0.710		-0.84	0.18	-1.188 - -0.500	
**LIGHT * SPECIES**				**<0.001*** **				**0.016***				0.341				0.259
*LIT * Leucanthemum vulgare*	0.027	0.15	-0.260 - 0.315		0.076	0.15	-0.211 - -0.363□		0.296	0.4	-0.485 - 1.077		-0.47	0.29	-1.033 - 0.096	
*LIT * Malva moschata*	0.734	0.19	0.361 - 1.106		0.52	0.19	0.159 - 0.882		-0.333	0.39	-1.093 - 0.428		-0.25	0.27	-0.778 - 0.288	

[1] SE = Standard Error, CI = Confidence
[2] *p<0.05; **p<0.01; ***p<0.001
